# Exploring the Process of Professional Role Redefinition Towards Recovery-Oriented Care Through Joint Crisis Plans in Japan: A Qualitative Study Using the Modified Grounded Theory Approach

**DOI:** 10.3390/healthcare14081003

**Published:** 2026-04-11

**Authors:** Mikie Ebihara, Tatsuya Tamura, Neteru Masukawa, Tomoko Omiya, Kumiko Ando

**Affiliations:** 1 Department of Nursing, School of Medicine, The Jikei University, 8-3-1 Kokuryo-cho, Chofu-shi, Tokyo 182-8570, Japan; 2 Faculty of Nursing, Fukushima Medical University, Hikarigaoka, Fukushima-shi, Fukushima 960-1295, Japan; 3 Inuyama Hospital, 10 Onoze, Toanochi, Aichi, Inuyama 484-0094, Japan; 4 Department of Public Health Nursing, Division of Health Science, Graduate School of Medicine, Tohoku University, 2-1 Seiryo-machi, Aoba-ku, Sendai 980-8575, Japan; toomiya-tky@umin.ac.jp (T.O.);; 5 Institute of Science Tokyo, Student Healthcare Center, 1-5-45 Yushima, Bunkyo-ku, Tokyo 113-8510, Japan

**Keywords:** joint crisis plan, recovery-oriented care, psychiatric advance directive, Modified Grounded Theory Approach, symbolic interactionism, deinstitutionalization, Japan, mental health nursing, positive risk-taking, shared decision-making

## Abstract

**Highlights:**

**What are the main findings?**

- JCP collaboration is associated with a three-stage process of professional role redefinition—from manager to recovery companion—across diverse psychiatric facility types in Japan.
- Co-construction of a shared language through JCP is associated with supporting conditions for individual autonomy, alleviating professional ethical dilemmas, and contributing to shifts in team-level care practices—from managerial to recovery-oriented practices.

**What are the implications of the main findings?**

- Within Japan’s structurally entrenched inpatient system, JCP collaboration functions as a potential tool for redefining professional roles and navigating structural managerial barriers; and inpatient collaborative JCP may serve as a preparatory phase for supporting role redefinition towards recovery-oriented care.

**Abstract:**

**Background/Objectives**: Japan’s mental healthcare system is characterized by the world’s highest number of psychiatric beds, widespread “social hospitalization,” and a structurally entrenched managerial support model that frequently undermines patient autonomy. Joint Crisis Plans (JCPs)—collaboratively developed crisis management documents—have been increasingly adopted as a care coordination tool; however, their role in transforming professional practice towards recovery-oriented support remains underexplored. This study aimed to elucidate the experiences of professionals utilizing JCPs across diverse facility types and to develop a theoretical understanding of the process by which they redefine their role from ‘manager’ to ‘recovery companion’. **Methods:** A qualitative design using the Modified Grounded Theory Approach (M-GTA), grounded in symbolic interactionism, was employed. Semi-structured interviews were conducted with 13 professionals (7 nurses, 6 mental health and welfare workers) across nine facilities (psychiatric hospitals, 24-h residential facilities, outpatient facilities) in the Kanto region of Japan. Theoretical sampling continued until saturation. Data were analyzed using the constant comparative method, with validity ensured through team checking. **Results**: Nine categories and 23 subcategories were extracted. A three-stage support transformation process emerged: (1) Initial Stage and Motivation, in which professionals confronted the limitations of managerial practice; (2) Role Redefinition and Practice through Collaboration, involving joint crisis management, strength-based support, and network building; and (3) Stage of Integration of Perspectives and Implementation of Recovery-Oriented Practice, in which professionals witnessed individual recovery and integrated new support values into their practice. Negative cases revealed that JCP effectiveness is contingent on the co-construction of shared meaning rather than procedural compliance. **Conclusions**: JCP was suggested to function as a potential tool to facilitate navigating and reframing structural managerial barriers in Japanese mental healthcare. The creation of a shared language through JCP was associated with supporting conditions for individual self-determination, alleviating professional conflicts, and contributing to shifts in organizational culture.

## 1. Introduction

A Crisis Plan (CP) refers to a document outlining coping strategies predetermined by the individual for addressing critical situations such as symptom relapse in schizophrenia [1,2]. Plans developed collaboratively between the individual and professionals are termed Joint Crisis Plans (JCPs) and are utilized in settings such as inpatient facilities and home-visit nursing [3]. The CP originated as part of the WRAP self-help programme in the United States, developed in response to the limitations of deinstitutionalization [4]. Internationally, JCPs have become established as a form of psychiatric advance directive (PAD), legally safeguarding self-determination and forming the basis for multi-professional outreach [5,6,7,8]. International guidelines also recommend them as an alternative to physical restraints [8].

Japan’s mental healthcare system occupies a distinctive position relative to international trends in deinstitutionalization. It has the world’s highest number of psychiatric beds, with an average hospital stay of approximately 280 days [9]. Furthermore, ‘social hospitalization’—long-term stay for non-clinical reasons—affects approximately 70,000 individuals due to insufficient community resources [10,11]. Structural barriers perpetuating this phenomenon include restricted housing market access [12], limited availability of individually tailored employment support [13], and a scarcity of community-based crisis intervention services capable of managing psychiatric instability [14].

Within this context, coercive interventions are frequently employed, making the protection of patients’ human rights a critical challenge [15]. In the absence of established legal frameworks for advance decision-making, the Japanese version of the JCP has emerged as a care coordination tool; however, a risk-management-oriented care model continues to undermine patient autonomy [16,17,18]. This structural context generates significant role conflict for nursing staff and mental health welfare workers navigating between safety obligations and recovery-oriented values.

To reflect the continuum envisioned by Japan’s Community-based Integrated Care System policy, this study spans three facility types: inpatient, 24-h community, and outpatient settings. Building on Thornicroft et al. [8] and Farrelly et al. [19]—whose work highlighted clinician-side barriers in Western community contexts—the present study extends this literature by examining cross-facility professional experiences within Japan’s uniquely institutionalized psychiatric system.

This study aims to elucidate the experiences of professionals utilizing JCPs across diverse facility types within Japan’s structurally managerial mental health care system, and to develop a theoretical understanding of the process by which professionals redefine their role from ‘manager’ to ‘recovery companion’—an account that complements prior RCT evidence by explaining the relational and organizational mechanisms through which JCPs produce change. To this end, we employed the Modified Grounded Theory Approach (M-GTA).

## 2. Materials and Methods

### 2.1. Research Design

We employed the Modified Grounded Theory Approach (M-GTA) [20]. Unlike classical Grounded Theory, which often aims for highly abstract, universal theories, M-GTA is specifically tailored for human services research. Grounded in symbolic interactionism, we selected M-GTA for its capacity to capture dynamic interactions and meaning-making processes within a specific clinical context. This practical framework aligns with our objective: to elucidate how professionals reconstruct their identities through real-world collaborative practice.

### 2.2. Research Participants

Research participants comprised nurses and mental health and welfare workers with experience in creating and utilizing JCPs. Participation was not limited to nursing staff but encompassed professionals across multiple disciplines and facility types. Participants were restricted to professionals with experience supporting individuals diagnosed with schizophrenia who had been discharged from psychiatric hospitals and had maintained community living for over one year, working in settings including psychiatric hospitals and social rehabilitation facilities.

In accordance with the 2023 MHLW guidelines, this study defined the duration as one year or more. This threshold reflects the practical necessity of long-term “accompanying support” and addresses the high-risk period post-discharge, where readmission rates in Japan exceed 30% within the first year [11]. Focusing on this critical window allows for an examination of how JCPs serve as a potential tool for early crisis avoidance through the co-construction of a shared crisis language and the internalization of help-seeking behaviors.

### 2.3. Definition and Types of JCP in Japan

In Japan, the JCP is implemented as CP-J (Crisis Plan Japan) [21], a localized professional-led clinical tool [2]. It is utilized as outreach-based for community stabilization [3] or inpatient-based for discharge support [1,17]. Notably, in 24-h community support settings (home-visit nursing stations), ACT-based crisis planning is employed as a collaborative framework. While these delivery structures differ, they are analytically comparable as they share a treatment-integrated, collaborative ethos. In the M-GTA analysis, ACT-specific processes were analyzed as contextual variations, ensuring a consistent theoretical focus on professional role redefinition.

### 2.4. Data Collection

Data collection was conducted from May to September 2019. Recruitment was initially conducted through convenience sampling of psychiatric ward nurses; subsequently, aligned with Japan’s Community-based Integrated Care System policy, theoretical sampling was implemented to include multidisciplinary professionals (nurses and mental health and welfare workers) from 24-h residential and outpatient facilities until theoretical saturation was achieved. This ensured a comprehensive comparison of professional roles across the continuum of care. Semi-structured interviews used a guide that evolved through the constant comparative process, moving from JCP procedures to exploring professional actions, identity reconstruction, and changes in multidisciplinary collaboration. All interviews were conducted individually by the lead researcher and audio-recorded with participants’ informed consent; interview duration ranged from 30 to 70 min (mean approximately 42 min).

### 2.5. Analytical Method

To ensure analytical rigour, the multidisciplinary research team included psychiatrists, clinical experts in PADs and Joint Crisis Plans (JCPs), service users with lived experience actively advancing these practices, and a sociologist with expertise in M-GTA. Formal analysis was conducted collaboratively by five members of this team (M.E., T.O., K.A., N.M., and T.T.), who continuously cross-validated the analytical summaries and emergent concepts through rigorous peer debriefing. Where discrepancies in concept interpretation or category assignment arose, these were resolved through iterative discussion and consensus within the team rather than by the lead researcher alone. The lead researcher’s extensive prior clinical involvement with JCPs was explicitly acknowledged as a potential source of interpretive bias—specifically, the risk of predisposing interpretations towards affirming JCP effectiveness. To mitigate this, the lead researcher maintained a reflexive journal throughout analysis, and team members without direct JCP experience were tasked with challenging interpretations that appeared to reflect the researcher’s prior perspective rather than the data. All analytical decisions were documented in concept definition worksheets to ensure transparency and traceability. In practice, this process led to two notable revisions during analysis: an initial subcategory tentatively labelled ‘empowerment through JCP’ was reconceptualised as ‘co-constructing shared language’ after team members cautioned that the former presupposed positive outcomes; and a proposed causal link between Stage 2 and Stage 3 was qualified to an associative relationship following deliberation over the absence of disconfirming cases at that transition point. The three-stage structure became evident as recurring patterns of professional role transformation—from confronting structural barriers, through collaborative role redefinition, to integration of recovery-oriented values—were confirmed across all facility types. Participant feedback was solicited to assess the ecological validity of the theoretical model. Practitioners and service users with lived experience of the practices under study confirmed that the emerging model resonated with their clinical realities. These insights were subsequently incorporated into the final analytical iteration. The study adhered to the COREQ guidelines [22]; the completed checklist (Supplementary Material File S1) is available from the corresponding author upon request. Theoretical saturation, as applied in M-GTA, refers to the achievement of conceptual depth within the identified categories—specifically, the point at which no new properties or relational variations emerged through continued comparative analysis—rather than the exhaustive representation of all facility types or participant roles. Saturation was considered reached when the nine categories and their interrelationships showed no further conceptual elaboration across the data. We acknowledge that, with 13 participants, the depth rather than breadth of coverage is what is claimed, and that replication across broader samples remains warranted.

## 3. Results

### 3.1. Overview of Research Participants and Survey

The research collaborating facilities comprised nine facilities: three psychiatric hospitals in the Kanto region, one psychiatric visiting nursing station, one residential independent living training facility, one group home (GH), one employment support facility, and two community activity support centers. Facilities were categorized as: psychiatric hospitals providing inpatient treatment (R, S, T); 24-h facilities providing continuous residential or home-visit support (U, V, W)—comprising a psychiatric visiting nursing station (U) offering round-the-clock ACT-based outreach, a group home (V) providing 24-h on-site residential support, and an independent living training facility (W) offering comprehensive live-in rehabilitation; and outpatient facilities providing support through treatment programmes or daytime activities (X, Y, Z). The 13 participants comprised 7 nurses (A–G) and 6 mental health and welfare workers (H–M), all with at least five years of psychiatric experience, one to three years of JCP support experience, and one to three support cases. Interviews lasted 30–70 min (mean ≈ 42 min). Both outreach-type [3] and inpatient-type [17] JCPs were utilized, with development generally requiring one to three months. Ancillary psychoeducation was conducted individually (R, S) or in group sessions (T). The target population was broad, ranging from individuals with repeated admissions to those experiencing work-related stressors, suicidal ideation, or social isolation (Table 1).

### 3.2. Professional Experiences in JCP Collaboration

Nine categories and 23 subcategories were extracted. Three stages of recovery-oriented support emerged through JCP collaboration, characterized by confronting the limitations of conventional support and reorienting towards person-centered care: (1) Initial Stage and Motivation; (2) Role Redefinition and Practice through Collaboration; and (3) Stage of Integration of Perspectives and Implementation of Recovery-Oriented Practice (Table 2).

#### 3.2.1. Initial Stage and Motivation

This phase centers on recognizing limits and the emergence of motivation through confronting structural and clinical challenges.

##### Navigating Structural Risk-Management Pressure

Professionals grappled with institutional pressures of risk management while facing complex clinical and social crises.

[Confronting intervention attempts and refusals]:

In acute wards, intervention to prevent physical deterioration was at times impossible:

*“Even getting them to take their medication proved impossible without communication. Ultimately, after six months in a secure room, they developed urinary retention and pressure sores.”*

**(Participant F)**

[Recognition of crises stemming from the living environment]

Professionals identified daily life stressors as significant triggers for psychological and social crises.

*“Family conflicts, living expenses, and money were significant stressors.”*

**(Participant I)**

[Difficulty of supporting isolation]:

Caught between the family’s fear of past harm and the individual’s longing for reunion, professionals confronted the limits of family-based support.

*“For example, family members stating they never wished to see the individual again.”*

**(Participant F)**

[Heavy responsibility of protecting the lives of patients in crisis]:

Lacking access to community-based crisis intervention teams, non-medical staff bore a disproportionate burden of accountability for managing life-threatening risks.

*“We are in a position where the facility bears responsibility for the risk of loss of life.”*

**(Participant K)**

##### Dilemmas Regarding Autonomy and Crisis Awareness

Professionals experienced clinical dilemmas arising from the gap between individual autonomy and a lack of crisis awareness.

[Difficulty in self-recognition of crisis status]:

Professionals encountered a breakdown in help-seeking behaviors, as individuals prioritized social demands over health and lacked recognition of their clinical crisis.

*“They were the type who would say ‘I absolutely refuse to be admitted!’ and refuse hospitalization even when clearly unwell.” “They only thought about getting discharged quickly. Because they believed taking time off work meant losing their job, they stopped attending outpatient appointments—and their symptoms kept getting worse.”*

**(Participant D)**

[Lack of decision-making and autonomy]:

*Professionals observed a profound loss of agency, where individuals either resisted engagement or passively complied with protocols solely to secure discharge.*

*“They had a very strong resistance to initiating anything new… They had no sense of ‘this is what I want to become, this is what I want to do’.”*

**(Participant A)**

*“There was no refusal [of JCP initiatives]; they engaged in it almost transactionally, as a means to secure their discharge and return home.”*

**(Participant D)**

#### 3.2.2. Role Redefinition and Practice Through Collaboration

##### Co-Constructing a Shared Language Through JCP

Professionals redefined their practice from managerial intervention to collaborative support by co-constructing a “shared language” through the JCP process.

[Supporting confrontation with the crisis]:

Professionals empowered individuals to demonstrate their self-management capacities before a crisis escalated, providing an opportunity to restore trust and gain recognition from their support network.

*“I made a promise to my patient: ‘Let’s use the JCP to show everyone that you can handle things on your own before a crisis hits.’ It was a way to restore trust and let those around them see how much progress they had made.”*

**(Participant F)**

[Building collaborative relationships for crisis management]:

Professionals prioritized fostering a “shared language” through mutual dialogue over rigid protocols, grounding crisis management in a collaborative helping relationship.

*“Ultimately, it’s about the relationship. There are times when patients say, ‘No, go home!’, but instead of jumping straight into the JCP, we ask, ‘What exactly is troubling you?’ and think together… conversely, we often end up learning a lot from them as well.”*

**(Participant G)**

[Having a shared language]:

The JCP process underscored identifying deeper meanings in individuals with limited verbal communication.

*“For a patient whose verbal expressions were almost entirely limited to phrases such as ‘I want to die’ or ‘Oh no,’ a professional realized: ‘The “fun” I speak of might be fundamentally different from what the patient experiences as “fun.”’”*

**(Participant K)**

##### Recalibrating Professional Roles for Engagement

Professionals shifted their roles from clinical experts to partners in daily life, lowering communication barriers and bridging the gap between hospital and community settings.

[Adjusting ease of engagement and workload]:

Professionals intentionally reduced the psychological burden and clinical pressure to facilitate more natural interactions.

*“We don’t use difficult words. We don’t give them the pressure that they have to try hard.”*

**(Participant B)**

[Promoting an image of community living]:

Professionals facilitated individualized discharge and community integration following long-term hospitalization and social isolation.

*“We spent time awakening the ‘reality of life’ through conversation, such as how to shop for a single carrot. If we just say ‘you can be discharged now’ and throw them out after decades, they will be lost in a world that has changed too much”*

**(Participant A)**

*“Let them have a mobile phone, withdraw money from the bank, and manage it themselves.”*

**(Participant G)**

##### Reconstructing Patient Narratives for Clinical Insight

Professionals reframed clinical insights into collaborative narratives, prioritizing agency over correction to break negative cycles and foster personal identity.

[Rebuilding the way of dealing with the illness]:

To break the cycle of the patient’s problematic behavior and the resulting dismissive attitude from exhausted staff, the professional prioritized individual agency over a corrective approach…

*“To address the passive and dependent behavior that had exhausted the staff, I dared to support the unrealistic coping she had proposed by suggesting she ‘give it a try.’ The patient realized, ‘It doesn’t fit my reality, so I’ll rewrite it,’ and she subsequently rewrote the plan herself.”*

**(Participant K)**

[Creating a new supportive relationship]:

This reformulation restructured the support relationship into a long-term partnership anticipating sustained goals.

*“We reframed the crisis as ‘to continue working’ and kept repeating it.”*

**(Participant M)**

[Reconstructing a life true to the individual]:

A professional described supporting an individual’s shift in focus—from symptom preoccupation towards actively pursuing what mattered to them.

*“It’s painful being envied and marginalized by the fan club members, but I still want to go see them because I love that artist. I’ll keep supporting them from a slight distance.”—The patient continued to attend.”*

**(Participant M)**

##### Fostering Collaborative Connections Against Isolation

Professionals supported the process of individuals reclaiming their community roles while managing their health, aiming to alleviate social isolation associated with their illness.

[Promoting social participation and alleviating isolation]:

*Once distanced by illness, they have learned to manage their own health and fulfill their roles within the community.*

*“Remarkably, he was able to discharge his responsibilities as chief mourner admirably.”* 

**(Participant B)**

[Cultivating a collaborative support network]:

The JCP served as evidence of the patient’s sincere commitment; even for the nurses who had given up on his recovery, it shifted the focus from risk management towards a positive acceptance of the individual.

*“By using the JCP to show how deeply the patient was reflecting on his recovery, I persuaded the staff to ‘take another chance’ on him; this redirected their interest towards his sincere efforts and fostered a growing atmosphere of support throughout the ward”.* 

**(Participant F)**

#### 3.2.3. Negative Case Analysis

To strengthen theoretical robustness, cases in which support transformation did not occur were examined. Two predominant patterns emerged: (a) denial of illness by the individual or family, which precluded psychoeducation and shared crisis understanding; and (b) procedural JCP completion driven by institutional requirements rather than the individual’s needs. A representative instance of the former was described by **Participant D**:

*“The individual insisted they had no illness and that the neighbors’ complaints were entirely the neighbors’ fault. The family likewise maintained that, since the individual denied being ill, there was no illness—and therefore no need for a JCP. During home visits, we were not even permitted to enter the house.”* 

**(Participant D)**

In both patterns, the JCP failed to generate a shared language, and individuals continued to cycle through crisis, compulsory intervention, and readmission—a ‘management loop’. This contrasts with successful cases, suggesting that JCP effectiveness is contingent not on the tool itself but on the quality of meaning co-construction during its creation, consistent with the symbolic interactionist framework of this study [16].

#### 3.2.4. Integration of Perspectives and Implementation of Recovery-Oriented Practice

Professionals integrated renewed recovery perspectives by witnessing individual progress in three areas: (1) restoration of patient agency, (2) co-creative ownership of the JCP, and (3) reframing of professional identity and care.

##### Witnessing the Restoration of Patient Agency

[Facilitating the recovery of self-esteem]:

The JCP fostered a shift from treatment avoidance to proactive help-seeking, facilitating the restoration of self-esteem.

*“They began taking notes in sessions and consulting their family, doctor, and even their employer. This culminated in them independently seeking care, saying: ‘I felt unwell and thought I needed to talk to someone.’”* 

**(Participant D)**

[Facilitating the elicitation of self-efficacy]

Professionals leveraged “shared language” to reinforce individual strengths, enabling patients to pursue independent living and employment goals by effectively managing interpersonal stress.

*“The patient has maintained employment for two years, aiming for independence from welfare. By using ‘shared language’ to process stress, they noted: ‘I no longer mind what people say [at work].’”*

**(Participant M)**

##### Co-Creative Ownership of the JCP

Professionals actively supported individuals in internalizing the JCP as their own tool rather than an externally imposed document.

[Nurturing independence and autonomy]:

This unfolded through interventions promoting independence and autonomy.

*“We make promises in the JCP, like ‘Let’s try our best until tomorrow’. But we keep those promises moment by moment.”* 

**(Participant G)**

[Support for taking ownership of JCP]:

The JCP appeared to serve as a tangible anchor, which participants described as supporting a shared trajectory of recovery and contributing to a sense of collaborative memory.

*“Ultimately, to foster the individual’s confidence, it would be beneficial if they could keep this [JCP], take it out, and look at it themselves to reaffirm it.”*

**(Participant A)**

##### Reframing Professional Identity and Care

[Mitigating professional dilemmas]:

A shift from administrative rules led to collaborative engagement.

*“There is no manual or medication that guarantees success; instead, I prioritize decid* *ing the course of action together with the patient, unhindered by administrative conventions.”*

**(Participant G)**

[Humanizing perspectives on individuals]:

Human-centered understanding shifted the tone of collaboration.

*“By focusing on understanding the individual, the atmosphere of multidisciplinary collaboration shifted: conferences that had previously stalled with labels such as ‘a troublesome person’ became notably warmer.”* 

**(Participant K)**

[Evolution of care practices]:

Individual and family insights reshaped organizational routines.

*“A patient’s realization that the ward’s medication schedule was incompatible with their post-discharge plans prompted a shift towards patient-centered care. This provided an opportunity for multidisciplinary teams to re-evaluate and revise established ward routines.”* 

**(Participant F)**

*“Observing the family treating the adult patient as a child, I advised them to interact with him as a ‘working adult son’; this led to the realization that building a collaborative team—inclusive of the family—is essential for supporting independence.”* 

**(Participant K)**

## 4. Discussion

### 4.1. Reframing the ‘Managerial Meaning World’ and Reconstruction Through Shared

Language

The following discussion is grounded exclusively in professionals’ accounts. Observations regarding individual recovery, self-determination, and autonomy reflect how professionals perceived and narrated service users’ experiences; they do not constitute direct evidence of service users’ own perspectives. Underlying the difficulties professionals faced was a profound disconnect between the individual’s ‘solitary world of meaning’ surrounding crises and the professionals’ ‘managerial world of meaning’. JCP collaboration professionals appeared to co-construct meaning grounded in symbolic interactionism [23] through a shared language. Specifically, participants described the fulfilment of ‘small promises’ within the JCP as supporting individual autonomy. Through this process, participants appeared to develop a sense of ‘I can do it’ as an ‘intersubjective reality’ [24]—one in which the accumulation of kept promises generates a shared relational memory, linking individuals and professionals into a common world of meaning. This process aligns with supported decision-making frameworks that emphasize collaborative meaning construction as foundational to self-determination in mental healthcare [25].

The following interpretations of shifts in individual experience—including patients’ sense of agency and recovery—are inferred from professional accounts only, and should not be taken as direct evidence of service users’ subjective experience.

### 4.2. Situating Qualitative Findings Within the Broader Evidence Base

The RCT by Thornicroft et al. [8] and the qualitative investigations of Farrelly et al. [19] and Werning et al. [26] together constitute complementary evidence base for understanding how JCPs function in practice. The RCT demonstrated that JCPs did not significantly reduce compulsory admissions in a UK community mental health context—an important finding that raises questions not about whether JCPs work, but about how and under what conditions they produce change. It is precisely this process-level question that qualitative inquiry is positioned to address. The present study contributes to this complementary body of evidence by providing a theoretically grounded account, from a non-Western institutional context, of the relational and organizational conditions under which JCP collaboration is associated with professional role redefinition.

Our negative case analysis aligns with the broader pattern emerging from this evidence base: JCPs appear most meaningful when co-constructed as shared language rather than completed as administrative requirements. Structural barriers, power imbalances, and the quality of clinician engagement—identified by Farrelly et al. [19] and Werning et al. [26] in Western settings—emerged comparably in the present findings within Japan’s uniquely institutionalized context. We acknowledge that this study cannot establish causal efficacy of JCPs; rather, it illuminates the relational and organizational processes through which JCPs may—or may not—support change in professional practice, thereby complementing outcome-focused research with process-level understanding.

### 4.3. Sample Composition and the Structural Absence of Physician Involvement

The preponderance of nurses and mental health welfare workers among participants—with limited physician representation—reflects a structural reality of Japanese clinical practice: JCP creation is led primarily by nursing and welfare professionals in daily living support contexts, while active physician involvement remains rare. This asymmetry is theoretically significant. Physicians function as the apex of the managerial hierarchy and bear ultimate responsibility for risk management; a collaborative tool such as the JCP, which redistributes interpretive authority over crises, is structurally prone to conflict with this vertical authority gradient. The present findings therefore suggest that professional role redefinition arises first among those most embedded in individuals’ daily lives, and that this ground-level shift may subsequently contribute to broader organizational culture change.

### 4.4. The Shift from Risk Management to ‘Positive Risk-Taking’

Within nursing in particular, the JCP functioned as a ‘bridge between risk and recovery’, connecting the profession’s robust duty of care with recovery-oriented principles. Avoiding dehumanizing, containment-oriented managerial interventions and reframing crises as ‘challenges to be overcome’ through the lens of positive risk-taking [27] represents a significant departure from Japan’s prevailing management model. Through this process, professionals redefined their role from ‘managers’ to support persons who uphold the autonomy of those in their care. This identity shift is particularly significant for the most resistant cases.

### 4.5. Shifts in Multidisciplinary Collaboration Dynamics

The impact of JCP extended beyond individual support, contributing to the construction of a ‘shared purpose’ across multidisciplinary teams. The JCP, functioning as a shared physical tool and common language, appeared to support organizational renewal of perspectives and reduction in stigma, potentially contributing to shifts in what had previously been a managerial setting towards a ‘warm conference’ culture.

## 5. Limitations

Several limitations of this study warrant acknowledgment. First, the sample comprised 13 professionals recruited from facilities within the Kanto region of Japan, which may limit the transferability of findings to other geographic and institutional contexts within Japan and internationally. Second, data collection was conducted in 2019, prior to the COVID-19 pandemic, which significantly restructured psychiatric service delivery and accelerated remote care models; the extent to which the extracted transformation process remains applicable to the current landscape requires further investigation. Third, the sample included no psychiatrists, reflecting a structural reality of Japanese JCP practice rather than a sampling limitation per se; nevertheless, the absence of physician perspectives constrains the generalizability of findings regarding multidisciplinary team dynamics. Fourth, two participants (G and H) utilized ACT-based crisis planning rather than CP-J, introducing heterogeneity in the intervention examined; while both share a collaborative, person-centered ethos, their structural differences were addressed analytically as contextual variations, and future studies should examine these frameworks separately. Fifth, as the primary researcher conducted all interviews and led the analysis, reflexivity is an inherent concern; the lead researcher’s prior JCP involvement may have predisposed interpretations towards affirming their effectiveness. While M-GTA does not require formal inter-coder reliability (ICR)—relying instead on team-based peer debriefing, reflexive journaling, and documented concept definition worksheets—the absence of independent coding verification is acknowledged as a limitation. Sixth, while theoretical saturation was confirmed by the absence of new conceptual variations across all three facility types, saturation achieved with 13 participants across diverse settings should be interpreted with caution; replication with larger and more geographically diverse samples is warranted. Seventh, this study collected no data from service users; all findings related to individual recovery and self-determination reflect professional perspectives only and should be interpreted accordingly. Future research incorporating service user perspectives is needed.

## 6. Conclusions

This study elucidated how JCP collaboration was associated with a progressive redefinition of professional roles—from ‘manager’ to ‘recovery companion’—within Japan’s structurally managerial psychiatric settings. While the scope of this study does not permit system-level conclusions, the extracted process may inform the design of future interventions and policies at the facility level.

The collaborative JCP process during hospitalization may function as a meaning co-construction framework, incrementally cultivating the conditions for genuine self-determination that PAD alone cannot provide.

Further analysis is required of the interaction between professional transformation and the subjective experience of individuals regaining self-control. Validation is also needed for collaborative models in which peer supporters function as ‘potential tools’ for renewing professionals’ rigid risk perceptions [28].

At the institutional level, future research and policy development may benefit from conceptualizing a developmental continuum—from inpatient collaborative JCP through to community-based recovery support—rather than treating these as parallel or competing frameworks.

## Figures and Tables

**Table 1:** Characteristics of research participants and collaborating facilities.

Research Participant	A	B	C	D	E	F	G	H	I	J	K	L	M
Profession	Nurse	Nurse	Nurse	Nurse	Nurse	Nurse	Nurse	Psychiatric Social Worker	Psychiatric Social Worker	Psychiatric Social Worker	Psychiatric Social Worker	Psychiatric Social Worker	Psychiatric Social Worker
age/gender	30+	30+	20+	40+	30+	40+	50+	30+	30+	30+	40+	50+	50+
Affiliated Facility	R	R	R	S	S	T	U	U	V	W	X	Y	Z
Facility Category	Psychiatric Ward	Psychiatric Ward	Psychiatric Ward	Psychiatric Ward	Psychiatric Ward	Psychiatric Ward	Psychiatric Home-visit Nursing Station	Psychiatric Home-visit Nursing Station	Group Home	Rehabilitation Facility for Indep. Living	Community Activity Support Center	Community Activity Support Center	Employment Support Facility
Type of Crisis Plan	CP-J	CP-J	CP-J	CP-J	CP-J	CP-J	ACT	ACT	CP-J	CP-J	CP-J	CP-J	CP-J
Facility Type	Inpatient Treatment Facility	Inpatient Treatment Facility	Inpatient Treatment Facility	Inpatient Treatment Facility	Inpatient Treatment Facility	Inpatient Treatment Facility	24-h Facility	24-h Facility	24-h Facility	24-h Facility	Outpatient Facility	Outpatient Facility	Outpatient Facility
Interview Duration (min)	40	40	40	30	30	60	50	40	30	40	40	40	70

**Table 2:** Categories and subcategories of professional experiences in JCP collaboration, stratified by facility type.

Support Phase	Category	Subcategory
**Initial Stage and Motivation**	Navigating structural risk-management pressure	Confronting intervention attempts and refusals
		Recognition of crises stemming from the living environment
		Difficulty of supporting isolation
		Heavy responsibility of protecting the lives of patients in crisis
	Dilemmas Regarding Autonomy and Crisis Awareness	Difficulty in self-recognition of crisis status
		Lack of decision-making and autonomy
**Role Redefinition and Practice through Collaboration**	Co-constructing a shared language through JCP	Supporting confrontation with the crisis
		Building collaborative relationships for crisis management
		Having a shared language
	Recalibrating professional roles for engagement	Adjusting ease of engagement and workload
		Promoting an image of community living
	Reconstructing patient narratives for clinical insight	Rebuilding the way of dealing with the illness
		Creating a new supportive relationship
		Reconstructing a life true to the individual
	Fostering Collaborative Connections Against Isolation	Promoting social participation and alleviating isolation
		Cultivating a collaborative support network
**Integration of Perspectives and Implementation of Recovery-Oriented Practice**	Witnessing the restoration of patient agency	Facilitating the recovery of self-esteem
		Facilitating the elicitation of self-efficacy
	Co-Creative Ownership of the JCP	Nurturing independence and autonomy
		Support for taking ownership of JCP
	Reframing Professional Identity and Care	Mitigating professional dilemmas
		Humanizing perspectives on individuals
		Evolution of care practices

## Data Availability

The data presented in this study are not publicly available due to ethical restrictions protecting participant privacy and anonymity.

## Supplementary Materials

The following supporting information can be downloaded at: https://www.mdpi.com/article/10.3390/healthcare14081003/s1, File S1: COREQ Checklist.

## Abbreviations

JCP: Joint Crisis Plan; PAD: Psychiatric Advance Directive; M-GTA: Modified Grounded Theory Approach; CP: Crisis Plan; ACT: Assertive Community Treatment; CP-J: Crisis Plan Japan version; COREQ: Consolidated Criteria for Reporting Qualitative Research; WRAP: Wellness Recovery Action Plan; RCT: Randomized Controlled Trial.
